# Perspectives in Immunotherapy: meeting report from the Immunotherapy Bridge, December 1st–2nd, 2021

**DOI:** 10.1186/s12967-022-03471-y

**Published:** 2022-06-07

**Authors:** Paolo A. Ascierto, Antonio Avallone, Nina Bhardwaj, Carlo Bifulco, Sergio Bracarda, Joshua D. Brody, Luigi Buonaguro, Sandra Demaria, Leisha A. Emens, Robert L. Ferris, Jérôme Galon, Samir N. Khleif, Christopher A. Klebanoff, Tamara Laskowski, Ignacio Melero, Chrystal M. Paulos, Sandro Pignata, Marco Ruella, Inge Marie Svane, Janis M. Taube, Bernard A. Fox, Patrick Hwu, Igor Puzanov

**Affiliations:** 1 Department of Melanoma, Cancer Immunotherapy and Innovative Therapy, Istituto Nazionale Tumori IRCCS “Fondazione G. Pascale”, Naples, Italy; 2 Experimental Clinical Abdominal Oncology Unit, Istituto Nazionale Tumori IRCCS “Fondazione G. Pascale”, Naples, Italy; 3 Tisch Cancer Institute, Icahn School of Medicine at Mount Sinai, New York, NY USA; 4 Providence Genomics and Earle A. Chiles Research Institute, Portland, OR USA; 5 Medical and Translational Oncology Unit, Department of Oncology, Azienda Ospedaliera Santa Maria, Terni, Italy; 6 Department of Medicine, Hematology and Medical Oncology, Tisch Cancer Institute, Icahn School of Medicine at Mount Sinai, New York, NY USA; 7 Department of Experimental Oncology, Innovative Immunological Models Unit, Istituto Nazionale Tumori IRCCS “Fondazione G. Pascale”, Naples, Italy; 8 Department of Radiation Oncology, Weill Cornell Medical College; Sandra and Edward Meyer Cancer Center; Department of Pathology and Laboratory Medicine, Weill Cornell Medical College, New York, NY USA; 9 Magee Women’s Hospital/UPMC Hillman Cancer Center, Pittsburgh, PA USA; 10 UPMC Hillman Cancer Center, Pittsburgh, PA USA; 11 INSERM, Laboratory of Integrative Cancer Immunology/Equipe Labellisée Ligue Contre Le Cancer/Centre de Recherche Des Cordeliers, Sorbonne Université, Université Paris Cité, Marseille, France; 12 The Loop Immuno Oncology Laboratory, Georgetown University Medical School, Washington, DC USA; 13 Human Oncology and Pathogenesis Program, Immuno-Oncology Service, Memorial Sloan Kettering Cancer Center (MSKCC)/Center for Cell Engineering, MSKCC/Parker Institute for Cancer Immunotherapy/Weill Cornell Medical College, New York, NY USA; 14 Head of New Therapeutic Products - Personalized Medicine, Lonza Global, Houston, TX USA; 15 Department of Immunology and Immunotherapy, Clinica Universidad de Navarra and CIBERONC, Pamplona, Spain; 16 Winship Cancer Institute at Emory University, Atlanta, GA USA; 17 Department of Urology and Gynecology, Istituto Nazionale Tumori IRCCS “Fondazione G. Pascale”, Naples, Italy; 18 Center for Cellular Immunotherapies and Division of Hematology-Oncology, University of Pennsylvania, Philadelphia, PA USA; 19 National Center for Cancer Immune Therapy (CCIT-DK), Department of Oncology, Copenhagen University Hospital, Herlev, Denmark; 20 Department of Dermatology, Johns Hopkins University SOM, Baltimore, MD USA; 21 Earle A. Chiles Research Institute, Robert W. Franz Cancer Research Center, Providence Cancer Institute, Portland, OR USA; 22 Moffitt Cancer Center, Tampa, FL USA; 23 Department of Medicine, Roswell Park Comprehensive Cancer Center, Buffalo, NY USA

**Keywords:** Immunotherapy, Checkpoint inhibitors, Combination therapy, Biomarkers, Tumor microenvironment, Vaccine

## Abstract

Over the past decade, immunotherapy has become an increasingly fundamental modality in the treatment of cancer. The positive impact of immune checkpoint inhibition, especially anti-programmed death (PD)-1/PD-ligand (L)1 blockade, in patients with different cancers has focused attention on the potential for other immunotherapeutic approaches. These include inhibitors of additional immune checkpoints, adoptive cell transfer (ACT), and therapeutic vaccines. Patients with advanced cancers who previously had limited treatment options available may now benefit from immunotherapies that can offer durable responses and improved survival outcomes. However, despite this, a significant proportion of patients fail to respond to immunotherapy, especially those with less immunoresponsive cancer types, and there remains a need for new treatment strategies.

The virtual Immunotherapy Bridge (December 1st–2nd, 2021), organized by the Fondazione Melanoma Onlus, Naples, Italy in collaboration with the Society for Immunotherapy of Cancer addressed several areas of current research in immunotherapy, including lessons learned from cell therapies, drivers of immune response, and trends in immunotherapy across different cancers, and these are summarised here.

## Introduction

Immunotherapy has become an increasingly fundamental modality in the treatment of cancer since immune checkpoint inhibitors were first approved for the treatment of advanced melanoma in 2011. Durable responses and improved survival can now be achieved with immune checkpoint blockade, especially with anti-programmed death (PD)-1/PD-ligand (L)1 inhibitors, in patients with many different cancers. However, while many patients with advanced cancer who previously had limited treatment options benefit from immunotherapy, a significant proportion fail to respond, especially those with less immunoresponsive cancer types. The need for new treatment strategies for these patients has focused attention on the development of other immunotherapeutic approaches, which include inhibitors of other immune checkpoints, adoptive cell transfer (ACT), and therapeutic cancer vaccines, as well as different treatment combinations.

The virtual Immunotherapy Bridge (December 1st–2nd, 2021), organized by the Fondazione Melanoma Onlus, Naples, Italy in collaboration with the Society for Immunotherapy of Cancer addressed several areas of current research in immunotherapy, including lessons learned from cell therapies, drivers of immune response, and trends in immunotherapy across different cancers, and these are summarised in this report.

## Lessons learned from cell therapies

### Enzyme CD26 activity augments CAR T cell therapy

CD26 is a surface glycoprotein expressed on various cell types including T cells, with CD26 + T cells associated with increased antitumor immunity. Three human CD4 + T cell subsets can be recognised based on their CD26 expression: one with regulatory properties (CD26^neg^), one with a naïve/central memory phenotype (CD26^int^), and one with a durable stem memory profile (CD26^high^). CD4 + CD26^high^ T cells have distinct antitumor and molecular properties relative to other helper subsets, producing increased levels of several cytokines including interleukin (IL)-17A, interferon (IFN)-γ, IL-22, and IL-2, with these cytokines being co-secreted. These properties suggest CD4 + CD26^high^ T cells may have potential as an immunotherapeutic approach.

CD26^high^ T cells mediate robust tumor immunity. In a murine mesothelioma model, CD26^high^ T cells eradicated tumors, while T helper (Th) 1 cells only regressed tumors short-term. Mice treated with CD26^high^ T cells survived significantly longer and this was associated with higher CD4 + and CD8 + chimeric antigen receptor (CAR) T cell engraftment and persistence compared with other T cell subsets [1]. CD4 + CD26^high^ T cells redirected with CAR had enhanced functional and antitumor activity versus classic human subsets (Th1, Th2, and Th17) or unselected CD4 + T cells.

Further evidence of a potential immunotherapeutic role is provided by a strong trend towards more CD26^high^ tumor-infiltrating lymphocytes (TILs) being observed in patients with oral cavity squamous cell carcinoma who responded to neoadjuvant PD-1 therapy [2, 3]. Clinical trials are now underway to evaluate the potential of CD4 + CD26^high^ T cells as next-generation ACT therapy in patients non-responsive to checkpoint blockade.

### Targeting solid malignancies with public neoantigen-specific T cell receptors

Although most cancer neoantigens are patient-specific with a high potential for both clonal heterogeneity and immunologic selection pressure, some may be shared across individuals. Oncogenic driver mutations can systematically reappear across patients, typically occurring in tightly constrained ‘hotspot’ regions within a protein. Moreover, these driver mutations often occur early in cellular transformation and contribute to maintaining a malignant phenotype; consequently, they tend to be clonally conserved both within a tumor mass and across metastases. Thus, a peptide containing a hotspot mutation bound by a relatively common human leukocyte antigen (HLA) allele could result in a ‘public’ neoantigen shared across many patients that is less susceptible to selection pressure [4].

*PIK3CA* is among the most common mutated driver oncogenes in human solid cancers [5]. *PIK3CA* substitutions occur in a three sets of hotspot regions with the most prevalent being in the protein’s kinase domain at the H1047 position.

A functional immunopeptidomic screen using monoallelic cell lines expressing individual HLA-A alleles and either mutant or wild-type *PIK3CA* was developed, with peptide/HLA-I complexes undergoing immune precipitation/liquid chromatography tandem mass-spectrometry (MS/MS). Analysis revealed PI3Kα-derived amino acid sequences exclusively from cells that co-express HLA-A*03:01 and mutant *PIK3CA*. Deconvolution of the MS/MS spectrum revealed a single nine amino acid peptide with a leucine for histidine substitution at the second position. This mutant peptide had a profoundly extended half-life compared with the wild-type epitope at physiological temperatures consistent with the substitution of a preferred HLA anchor residue.

Immunogenicity of this *PIK3CA* mutation was assessed in a subset of patients with cancer who express HLA-A*03:01. This was enabled using the Memorial Sloan Kettering-Integrated Mutation Profiling of Actionable Cancer Targets (MSK-IMPACT) clinical next-generation sequencing (NGS) platform which allows detection of somatic tumor alterations and germline encoded HLA-I alleles. Fourteen patients with different tumor types commonly associated with mutant *PIK3CA* were identified and underwent immune monitoring. Circulating *PIK3CA* public neoantigen-specific T cells were identified in four of these patients, but in none of five concurrently tested HLA-A*03:01 expressing healthy donors.

A series of unique T cell receptor (TCR) sequences that recognize this *PIK3CA* public neoantigen were cloned into retroviral expression vectors and used to infect otherwise non-specific CD4^+^ and CD8^+^ T cells. In the context of HLA-A*03:01, all TCR members exhibited absolute specificity for mutated *PIK3CA* with no responses detected against wild-type *PIK3CA*. When expressed in CD8^+^ T cells, two TCR sequences triggered significantly greater mutant-specific cytokine production and degranulation compared with two other TCRs. These TCRs also triggered T cell function when transduced into CD4^+^ T cells, indicating coreceptor independence. All four TCRs mediated cytolysis of mutant *PIK3CA*^+^/HLA-A*03:01^+^ target cells but not of wild-type *PIK3CA*/HLA-A*03:01^+^ cells. One TCR (TCR4) exhibited superior cytolytic efficacy relative to all other TCR library members. TCR4 contains a CDR3β loop that is significantly extended and its unique structural properties allow it to form an interface that extends across nearly the entire peptide length. This configuration likely contributes to the receptor’s relatively high affinity and specificity for the *PIK3CA* public neoantigen.

**Fig. 1:**
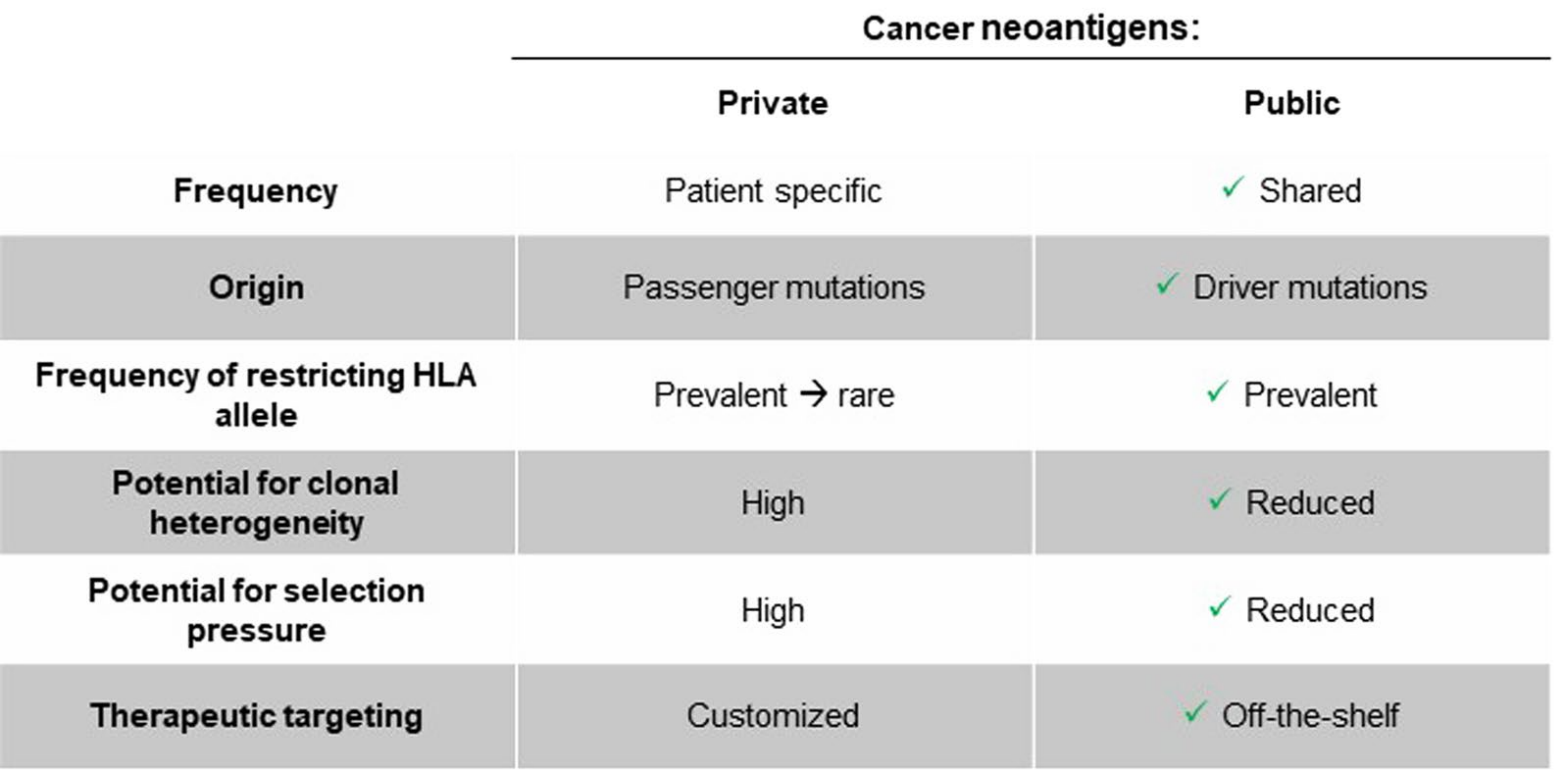
‹‹Private›› versus ‹‹Public›› neoantigens

Public neoantigens, including the newly described PIK3CA public neoantigen [6], therefore represent an innovative new translational platform to help develop TCR immunotherapies for patients [7] (see Fig. 1).

### Harnessing the power of a natural killer: bringing natural killer cells to immuno-oncology

CAR T cell therapies have revolutionized immunotherapy but autologous approaches have a number of challenges that hinder implementation, including interpatient variability, complex manufacturing processes, and high costs. Allogeneic models use third-party cell sources that are engineered to address the unique challenges of each cancer. Initial allogeneic strategies have focused on overcoming T-cell alloreactivity to produce universal CAR T cells. However, graft-versus-host disease (GvHD) and severe toxicities, namely cytokine release syndrome (CRS) and immune effector cell-associated neurotoxicity syndrome (ICANS) are concerns. Other immune effector cells have also been considered as potential candidates for cell therapy, including natural killer (NK) cells. Viable allogeneic NK cell sources include peripheral blood, umbilical cord blood, induced pluripotent stem cells, hematopoietic stem/progenitor cells and NK cell lines (NK-92). NK cell-focused approaches offer HLA-independent antigen recognition, an ability to modulate immune response and high cytotoxic potential, with a diverse cytotoxicity profile and enhanced cytotoxicity through antibody-dependent cellular cytotoxicity (ADCC). Moreover, NK cells are amenable to genetic manipulation, allow enhanced function by CAR expression, expand well in vitro*,* and are associated with a low risk of GvHD with no CRS or ICANS. In 11 patients with relapsed or refractory CD19-positive non-Hodgkin's lymphoma (NHL) or chronic lymphocytic leukemia (CLL), NK cells modified to express an anti-CD19 CAR resulted in eight responses [8]. Of these eight patients, seven had complete remission. Administration was not associated with the development of CRS, ICANS or graft-versus-host disease. CAR NK cells expanded and persisted at low levels for at least 12 months.

Cytokine-inducible SH2-containing protein (CIS), encoded by *CISH*, is a critical negative regulator of IL-15 signaling in NK cells and a potent checkpoint in NK cell-mediated tumor immunity [9]. *CISH*-knockout NK cells exhibit improved expansion and increased anti-tumor cytotoxicity [10]. Loss of *CISH* expression enhanced potency of cord-blood derived CAR-engineered NK cells in a lymphoma mouse model. As such, a strategy of CIS checkpoint deletion with CAR engineering may promotes activity of NK cells in an otherwise suppressive tumor microenvironment (TME) [11].

### Understanding resistance to develop better CAR T immunotherapies

Despite the success of CAR T therapies to treat B cell hematological malignancies, over half of patients still experience primary resistance or relapse [12]. Failures of CAR T immunotherapy can involve pre-infusion barriers, such as low lymphocyte counts, manufacturing failure, or disease progression occurring in parallel with the manufacturing process, as well as resistance related to CAR T cell dysfunction, an immunosuppressive TME, or tumor-intrinsic mechanisms [13]. CAR T cell dysfunction can include defective effector function due to an exhausted T cell phenotype, lack of expansion, or lack of persistence. Factors relating to the TME include impaired trafficking, metabolism/hypoxia, and the presence of immunosuppressive cells (stroma, myeloid cells, regulatory T cells) and cytokines (transforming growth factor [TGF]-β, IL-10, IL-35). Tumor-intrinsic mechanisms include loss of target antigen, expression of inhibitory ligands (e.g., PD-L1), lack of costimulatory ligands (e.g., CD58 loss), and resistance to immune killing.

Loss of target antigen has been the most widely studied mechanism of resistance [14]. In B cell acute lymphoblastic leukemia (B-ALL), CD19 loss accounts for up to 75% of relapses after CAR T cell therapy [15] while approximately a third of relapses in diffuse large B cell lymphoma (DLBCL) show CD19 loss or downregulation [16]. Strategies to overcome antigen loss are focused on targeting multiple antigens, which can be achieved using pooled CAR T cells, which involve different CAR T cells directed against different antigens, or through single T cells with multiple specificities (e.g., dual anti-CD19 and anti-CD22 CAR T cells). Fourth-generation CAR T cell have a single CAR but also secrete bispecific antibodies that target another antigen. In addition, CAR T cell therapy may be combined with a separate bispecific antibody.

One example of dual CAR T therapy is the co-administration of two humanized autologous CAR T cell products, one targeted to CD19 (huCART19) and the other to CD22 (CART22-65 s). In 13 patients with relapsed/refractory ALL, treatment with huCART19 and CART22-65 s resulted in complete remissions in 11 evaluable patients with minimal residual disease at one month [17]. Two patients developed toxicities before the 28-day assessment; one died from therapy-related complications, and one developed rapidly progressive disease. Another study showed mosunetuzumab, a bispecific antibody targeting CD20 and CD3, resulted in complete responses in some patients with heavily pretreated relapsed or refractory B-cell NHL and disease progression after CAR T cell therapy [18].

Lastly, Dr. Ruella discussed how the intestinal microbiome could modulate the anti-tumor immune response to ACT and, in particular, CART 19. In 228 patients with ALL or NHL treated with CD19 CAR T cells, the use of antibiotics, in particular piperacillin-tazobactam, imipenem-cilastatin, or meropenem (P-I-M), in the 4 weeks before therapy was associated with worse survival [19]. Moreover, the composition of the gut microbiota also correlated with outcomes. Indeed, increased abundance of *Lachnospiraceae, Ruminococcaceae,* and *Bacteroidaceae* spp. was observed in patients who achieved a complete response and those who experienced CAR-mediated toxicity.

In conclusion, resistance to CAR T immunotherapy can be driven by multiple factors [13]. Several therapeutic approaches are now being explored to overcome resistance, and, for example, initial results of targeting both CD19 and CD22 in ALL or combining ibrutinib and CAR T19 [20] are showing promising results.

### Best of SITC 2021 for clinical development and trials

BO-112 is a double-stranded synthetic RNA formulated polyethyleneimine that can mimic a viral infection and induce an immune response. In patients with advanced melanoma whose disease had progressed following anti-PD-1-based therapy, intratumoral administration of BO-112 in combination with pembrolizumab met the primary endpoint of the phase II SPOTLIGHT-203/KEYNOTE-B77 trial, with an objective response rate (ORR) of 27% in 37 evaluable patients [21]. Disease control rate (DCR) was 65% and responses were durable. The combination had a manageable safety profile with no patients discontinuing treatment due to adverse events. A further trial is planned.

In the NIVIPIT trial, intratumoral low dose ipilimumab in combination with nivolumab was associated with fewer grade ≥ 3 toxicities than intravenous (IV) ipilimumab with nivolumab in 61 previously untreated patients with metastatic melanoma [22]. Overall, 30% of patients receiving intratumoral ipilimumab 0.3 mg/kg had grade ≥ 3 treatment related adverse events versus 57% in the IV ipilimumab 3 mg/kg arm. No procedure-related ≥ grade 3 adverse events were reported in the intratumoral arm. ORR per Response Evaluation Criteria in Solid Tumors (RECIST) 1.1 were observed in 50% of patients in the intratumoral arm compared with 65% in the IV arm; 65.7% of tumors injected with ipilimumab showed a complete or partial response. The high response rate in injected lesions was associated with a reduction in intratumoral CD25^high^ CD39^high^ activated regulatory T cells (Tregs).

Vidutolimod is an immunostimulatory virus-like particle containing a CpG-A toll-like receptor 9 (TLR9) agonist. Promising clinical activity was observed with vidutolimod as monotherapy and in combination with pembrolizumab in patients with anti-PD-1-refractory melanoma in an open-label, phase Ib study [23]. Grade 3/4 treatment-related adverse events occurred in 37% of patients treated with vidutolimod plus pembrolizumab and in 22.5% of patients treated with vidutolimod monotherapy. No treatment-related deaths occurred. ORR was 23.5% with the combination, with 7.1% complete responses, and responses were durable (median duration of response [DOR] of 25 months). Vidutolimod also showed single agent activity, with an ORR of 20%. Clinical trials to confirm the efficacy of vidutolimod plus PD-1 blockade in patients with previously untreated unresectable/metastatic melanoma or PD-1 blockade-refractory melanoma are ongoing.

Electroporated plasmid IL-12 (pIL-12-EP) induces sustained intratumoral expression of IL-12, a cytokine involved in response to anti-PD-1 therapy. In updated data from the phase II KEYNOTE 695 trial of pIL-12-EP in combination with pembrolizumab, durable responses in both injected and non-injected lesions were observed in patients with stage III-IV melanoma with disease progression on anti-PD-1 therapy [24]. ORR per RECIST in 54 evaluable patients was 27.8%, with four complete and 11 partial responses. Seven patients had 100% reduction in target lesions, and regression was observed in non-injected lesions. Treatment was generally well tolerated, with minimal grade 3 and no grade 4–5 treatment-related adverse events.

Sotigalimab is a CD40 agonist antibody engineered to have increased binding to FcyRIIb. In a phase II trial, sotigalimab combined with nivolumab demonstrated tumor responses or prolonged disease control in 33 anti-PD-1/PD-L1-refractory melanoma patients with a favorable safety and tolerability profile [25]. Six patients had a partial response for an ORR of 18% and median DOR was 18.7 months. Three of the six responding patients remained off all therapy for ≥ 26 months. Three additional patients had prolonged stable disease. DCR was 48% and 33% of subjects experienced reduction in target lesions.

BNT111 is an RNA-lipoplex vaccine being assessed in the Lipo-MERIT trial, an ongoing, first-in-human, open-label, dose-escalation phase I study in pre-treated patients with advanced melanoma. In this analysis, promising signs of clinical activity were observed in patients with no evidence of disease at trial inclusion [26]. Median disease-free survival (DFS) was 34.8 months and T-cell immunity was induced irrespective of the presence of a clinically or radiologically detectable tumor. Immunogenicity and safety profiles were comparable to those previously reported for patients with evidence of disease at baseline.

BNT211 is a CAR T cell product candidate that targets the tumor-specific antigen Claudin-6 (CLDN6). Combination with a CAR T cell Amplifying RNA Vaccine (CARVac) leads to in vivo expansion of adoptively transferred CAR T cells, resulting in improved persistence and functionality. In a first-in human trial, CLDN6 CAR T cells with or without CARVac had a favorable safety profile and encouraging signs of efficacy in eight patients with advanced solid cancers [27]. No acute or dose-limiting toxicities and no serious adverse events related to the drug product were reported. Preliminary efficacy data showed initial tumor shrinkage according to RECIST 1.1 in all of five patients.

## Trends in immunotherapy

### Integrating head and neck cancer immunotherapy into locally advanced treatment

The first phase III study to show a benefit of immunotherapy in head and neck squamous-cell carcinoma (HNSCC) of the was the CheckMate-141 trial, in which treatment with nivolumab resulted in longer OS than treatment with standard, single-agent chemotherapy in patients with recurrent platinum-refractory disease [28, 29]. Subsequently, pembrolizumab improved overall survival (OS) as monotherapy versus cetuximab plus chemotherapy in patients with PD-L1-positive disease and in combination with chemotherapy versus cetuximab plus chemotherapy irrespective of PD-L1 status [30]. However, we still do not fully understand why some patients do not benefit or how to integrate with other existing modalities such as chemotherapy and radiotherapy.

In the locally advanced setting, avelumab plus concurrent chemoradiotherapy followed by avelumab maintenance did not improve progression-free survival (PFS) or OS versus chemoradiation alone in 697 patients with HNSCC in the JAVELIN 100 trial [31]. Sequential chemoradiation followed by pembrolizumab is being compared with concurrent chemoradiation and pembrolizumab in an ongoing trial in intermediate or high-risk patients. In another trial in intermediate-risk patients, pembrolizumab is being evaluated in combination with chemoradiotherapy and the ISA101b human papillomavirus (HPV) peptide vaccine in HPV-positive patients.

Transoral surgery can provide better access and control of surgical margins and selective neck dissection allows nodal treatment without significant additional morbidity. A recent trial investigated adjuvant de-escalated radiation plus concurrent nivolumab in 42 patients with intermediate-risk P16 + oropharyngeal cancer after transoral surgery and will report soon. Neoadjuvant immunotherapy is also being investigated in locally advanced HNSCC. In the CheckMate-358 trial, two doses of neoadjuvant nivolumab induced pathologic regressions in HPV-positive (23.5%) and HPV-negative (5.9%) tumours and was generally well tolerated with no unexpected delays to surgery due to adverse events in 52 patients with stage III–IV resectable HNSCC [32]. Survival is also improved after a neoadjuvant response; in analysis of data from two window trials in which patients received cetuximab or anti-ErbB3 antibody, patients with pathological downstaging migration had significantly improved 5-year OS and DFS compared to those without pathological downstaging [33].

The combination of neoadjuvant nivolumab plus ipilimumab has also been shown to be feasible, with higher response rates than with nivolumab monotherapy [34]. Several other neoadjuvant and adjuvant trials are ongoing, including the KEYNOTE-412 trial of pembrolizumab administered as a priming dose, concomitant with chemoradiation, and as maintenance therapy, as well as the KEYNOTE-689 trial of adjuvant and neoadjuvant pembrolizumab combined with chemoradiotherapy, and the HCC 18–139 trial which will compare neoadjuvant anti-PD-1 alone, anti-PD-1 plus anti-CTLA-4, and anti-PD-1 plus anti-lymphocyte activation gene (LAG)-3. Neoadjuvant approaches may help inform biomarker identification and more rational treatment combinations.

### Kidney cancer

Renal cell carcinoma (RCC) consists of different settings and subtypes with distinct molecular and histological tumor heterogeneity. Treatment options consequently range from an initial eventual possible observational phase in few good-risk patients and tyrosine kinase inhibitor (TKI) monotherapy or TKI/Immunotherapy combinations in the others, to immunotherapy combinations in intermediate/high-risk patients, especially those with sarcomatoid features, with TKI alone as a further treatment option.

In the CheckMate-214 trial of nivolumab plus ipilimumab versus sunitinib for first-line treatment of advanced RCC, combination immunotherapy was associated with improved PFS and OS which was maintained for up to 4 years, along with manageable safety [35]. OS remained superior with nivolumab and ipilimumab at 4 years in intermediate/high-risk patients but was not significantly improved in favorable-risk patients. These benefits were also sustained at 5-year follow-up, with 5-year OS rates of 43% with nivolumab plus ipilimumab versus 31% with sunitinib among intermediate/high-risk patients [36].

Proangiogenic factors can have immunomodulatory effects on the immune system, suggesting antiangiogenic therapy with vascular endothelial growth factor (VEGF) inhibitors combined with checkpoint inhibitors may have a synergistic effect. In the CheckMate-9ER trial, combination therapy with the multi-targeted TKI cabozantinib and nivolumab was superior to sunitinib monotherapy as first-line treatment for advanced RCC, with significantly longer PFS and OS [37]. Patients receiving the combination also had higher response rates than those treated with sunitinib monotherapy and health-related quality of life was significantly improved. Similarly, in the KEYNOTE-426 trial, pembrolizumab plus axitinib resulted in significantly longer PFS and OS as well as a higher ORR than sunitinib monotherapy in treatment-naïve patients with RCC [38]. Extended follow-up (median 30.6 months) continued to show higher efficacy of pembrolizumab plus axitinib with a 42-month OS rate of 57.5% versus 48.5% with sunitinib, and 42-month PFS rate of 25.1% versus 10.6% [39]. In a fourth trial, lenvatinib plus pembrolizumab was associated with significantly longer PFS and OS than sunitinib [40].

Partial or radical nephrectomy is frequently used for locoregional RCC but patients with intermediate/high-risk advanced disease are at risk of recurrence, and there are no standard treatment options after surgery to prevent relapse. However, adjuvant pembrolizumab following surgery significantly improved DFS at 2 years compared with placebo among patients with high-risk RCC in the phase III KEYNOTE-564 (77.3% vs. 68.1%; HR for recurrence or death, 0.68; 95% CI 0.53–0.87; p = 0.002) [41], Safety was consistent with that seen in other trials with no new safety signals.

### GI cancer

Esophageal cancer can be classified by histology as esophageal squamous cell carcinoma, which accounts for around 85% of cases, and esophageal adenocarcinoma. Therapeutic options are limited, independent of histological subtype, and prognosis remains poor with 5-year survival rates around 10% in Western countries. Nivolumab and pembrolizumab were first shown to improve OS with a favourable safety profile compared with chemotherapy as second-line treatment in heavily pretreated patients with advanced esophageal squamous cell carcinoma [42, 43]. Pembrolizumab in combination with chemotherapy also improved survival when given as a first-line option in patients with advanced and metastatic esophageal cancer, independent of histological subtype and PD-L1 status, in the phase III KEYNOTE-590 trial [44]. In the CheckMate-648 trial, first-line treatment with nivolumab plus chemotherapy or nivolumab plus ipilimumab improved survival versus chemotherapy alone in 970 patients with esophageal squamous cell carcinoma [45]. At a minimum follow-up of 13 months, both nivolumab plus chemotherapy and nivolumab plus ipilimumab resulted in significant improvements in median OS compared with chemotherapy alone in patients with tumor cell PD-L1 expression ≥ 1% and in all randomized patients. A significant PFS benefit was also observed for nivolumab plus chemotherapy versus chemotherapy in patients with tumor cell PD-L1 ≥ 1%, although PFS benefit did not meet the prespecified boundary for significance with nivolumab plus ipilimumab versus chemotherapy. Nivolumab-containing regimens had manageable safety profiles with no new safety signals. Significantly improved OS and PFS were also observed with the addition of the anti-PD-1 antibody camrelizumab to chemotherapy in 596 eligible patients with untreated advanced or metastatic esophageal squamous cell carcinoma in the ESCORT-1 trial [46].

In the CheckMate-649 trial, 1581 patients with previously untreated non-HER2-positive gastric, gastro-oesophageal junction, or esophageal adenocarcinoma, regardless of PD-L1 expression, were randomized to chemotherapy with or without nivolumab [47]. Nivolumab plus chemotherapy resulted in significant improvements in OS (hazard ratio [HR] 0.71; p < 0.0001) and PFS (HR 0.68; p < 0.0001) versus chemotherapy alone in patients with a PD-L1 combined positive score (CPS) of ≥ 5. OS and PFS benefit were also observed in all randomized patients. In longer-term follow-up, the addition of nivolumab to chemotherapy continued to produce a significant improvement in OS, with the greatest magnitude seen in patients with high PD-L1-expressing tumors. In contrast, the third arm of the study, in which patients received nivolumab and ipilimumab, did not significantly improve OS versus chemotherapy in patients with a PD-L1 CPS ≥ 5 [48]. These data support nivolumab plus chemotherapy as a new standard first-line treatment in patients whose tumors express PD-L1 with a CPS ≥ 5.

Immunotherapy is also being assessed in earlier settings for esophageal cancer. In CheckMate-577, nivolumab significantly improved DFS in patients with resected stage II/III esophageal or gastroesophageal junction cancer who had received neoadjuvant chemoradiotherapy and had residual pathological disease [49]. Several ongoing trials are also investigating PD-1/PD-L1 checkpoint inhibitors in the preoperative setting of locally advanced esophageal cancer.

In colorectal cancer, first-line therapy with pembrolizumab resulted in improved PFS versus chemotherapy in microsatellite-instability-high (MSI-H)-mismatch-repair-deficient (dMMR) metastatic colorectal cancer, with fewer treatment-related adverse events [50]. Ongoing trials are assessing PD-1 blockade in combination with chemotherapy and a VEGF inhibitor, and with ipilimumab. In the neoadjuvant setting, treatment with nivolumab in 22 patients with early-stage colon cancer was feasible, did not compromise surgery and led to an increase of TILs in the NICOLE trial [51]. Similarly, nivolumab plus ipilimumab was well-tolerated and all patients underwent surgery without delays in interim data from the NICHE trial [52]. Pathological response, complete or near complete, was observed in all 20 patients with dMMR tumors, while 4 of 15 patients with mismatch-repair-proficient tumors (27%) had a complete or near complete pathological response.

### Implications of the tumor microenvironment for breast cancer immunotherapy

In the phase III IMpassion 130 study, 902 patients with untreated locally advanced or metastatic triple-negative breast cancer (TNBC) were randomized to atezolizumab plus nab-paclitaxel or placebo plus nab-paclitaxel until disease progression or unacceptable toxicity. The addition of atezolizumab to nab-paclitaxel resulted in a statistically significant PFS benefit and clinically meaningful OS benefit versus nab-paclitaxel with placebo in patients who had PD-L1 immune cell-positive disease. At the final analysis, the HR for OS was 0.67 but statistical significance for OS was not formally tested as per the prespecified testing hierarchy [53]. Importantly, there was no treatment effect in patients whose tumors were PD-L1 immune cell-negative.

In a phase I study of atezolizumab monotherapy, improved clinical outcomes were observed in patients with tumors that were PD-L1 immune cell-positive, contained high levels of stromal TILs, or were of the basal-like immune-activated (BLIA) molecular subtype of TNBC [54]. Building on this data, an exploratory biomarker analysis of IMpassion 130 evaluating the association of PD-L1 expression on immune cells and tumor cells, intratumoral CD8, and stromal TILs was undertaken [55]. Intratumoral CD8 and stromal TIL positivity were associated with PD-L1 immune cell-positive status and predicted clinical benefit only in patients who were also PD-L1 immune cell-positive. In additional exploratory analyses, improved PFS was observed in patients with PD-L1 immune cell-positive disease with an immune-inflamed phenotype or immune-excluded phenotype, whereas improved OS was observed in only in patients with PD-L1 immune cell-positive immune-inflamed disease [56]. Improved PFS was also observed in patients with PD-L1 immune cell-positive tumors of the BLIA or basal-like immunosuppressed (BLIS) molecular subtype, whereas improved OS was observed only in patients with PD-L1 immune cell-positive of the BLIA molecular subtype. Potential mechanisms of resistance to PFS benefit among patients with PD-L1 immune cell-positive disease included LAR molecular subtype and tumors with increased angiogenesis, epithelial-mesenchymal transition signalling, hedgehog pathway signalling, estrogen response and tumor necrosis factor (TNF) pathway signalling. Potential mechanisms of resistance limiting OS benefit for patients with PD-L1 immune cell-positive disease included the BLIS and LAR molecular subtypes. All of these observations are hypothesis-generating and require follow up in independent data sets.

### Ovarian cancer

A meta-analysis of ten studies with 1815 ovarian cancer patients confirmed that TILs are a robust predictor of outcomes [57]. PD-L1 expression is associated with TILs and a favorable prognosis in high-grade serous ovarian cancer [58]. However, tumoral PD-L1 expression differs according to histologic type. BRCA 1/2 mutations result in homologous recombination deficient tumors and are associated with significantly higher CD8 + /CD4 + ratio of TILs and significantly higher peritumoral T cells and may be more susceptible to PD-1/L1 inhibition. BRCA1 and BRCA2 mutations have different effects on the TME and response to checkpoint immunotherapy. Thus, ovarian cancer is not a single disease and different immunophenotypes may require different treatment strategies.

Single agent checkpoint inhibitor studies in ovarian cancer have been generally disappointing, with response rates of around 10–20%. In a phase III study in 316 patients with platinum-resistant advanced or recurrent ovarian cancer, nivolumab did not improve OS compared with gemcitabine or pegylated liposomal doxorubicin [59]. PD-1 blockade in combination with chemotherapy has also been shown to be ineffective. In the JAVELIN Ovarian 200 trial, neither avelumab plus pegylated liposomal doxorubicin nor avelumab alone significantly improved PFS or OS versus pegylated liposomal doxorubicin alone in patients with platinum-resistant or refractory ovarian cancer [60]. Similarly, front-line chemotherapy with or without avelumab followed by avelumab maintenance did not improve outcomes versus chemotherapy alone in patients with previously untreated epithelial ovarian cancer in the JAVELIN Ovarian 100 trial [61].

A lack of PFS benefit from immunotherapy was also observed in the phase III IMagyn050 trial, in which newly diagnosed stage III or IV ovarian cancer patients were randomized to chemotherapy (paclitaxel plus carboplatin) plus bevacizumab with or without atezolizumab [62]. The addition of atezolizumab did not significantly improve PFS in the intent-to-treat population or in the PD-L1-positive (immune cells ≥ 1%) population. PFS benefits were seen with atezolizumab for patients with tumors with ≥ 5% PD-L1-positive immune cells, who represented approximately 20% of the overall study population. These findings may warrant further evaluation of atezolizumab in a population with high PD-L1 expression. There was also an improvement in PFS favoring atezolizumab for those with tumors having ≥ 1% PD-L1-positive tumor cells. Interim OS data showed no significant benefit with atezolizumab.

**Fig. 2:**
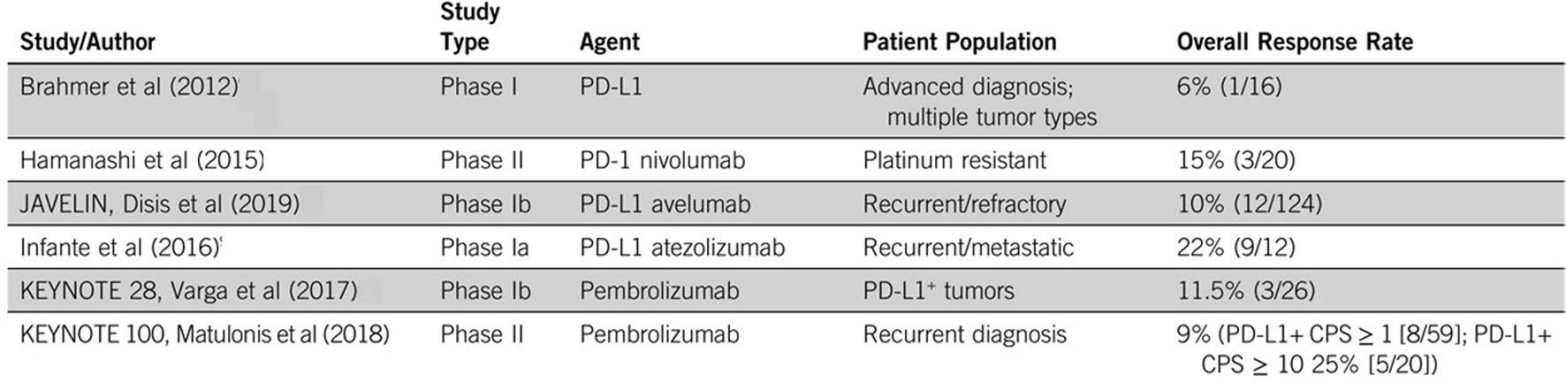
Single-agent checkpoint inhibitor stuides in ovarian cancer. Levinson et al. ASCO Ed. Book (2019)

**Fig. 3:**
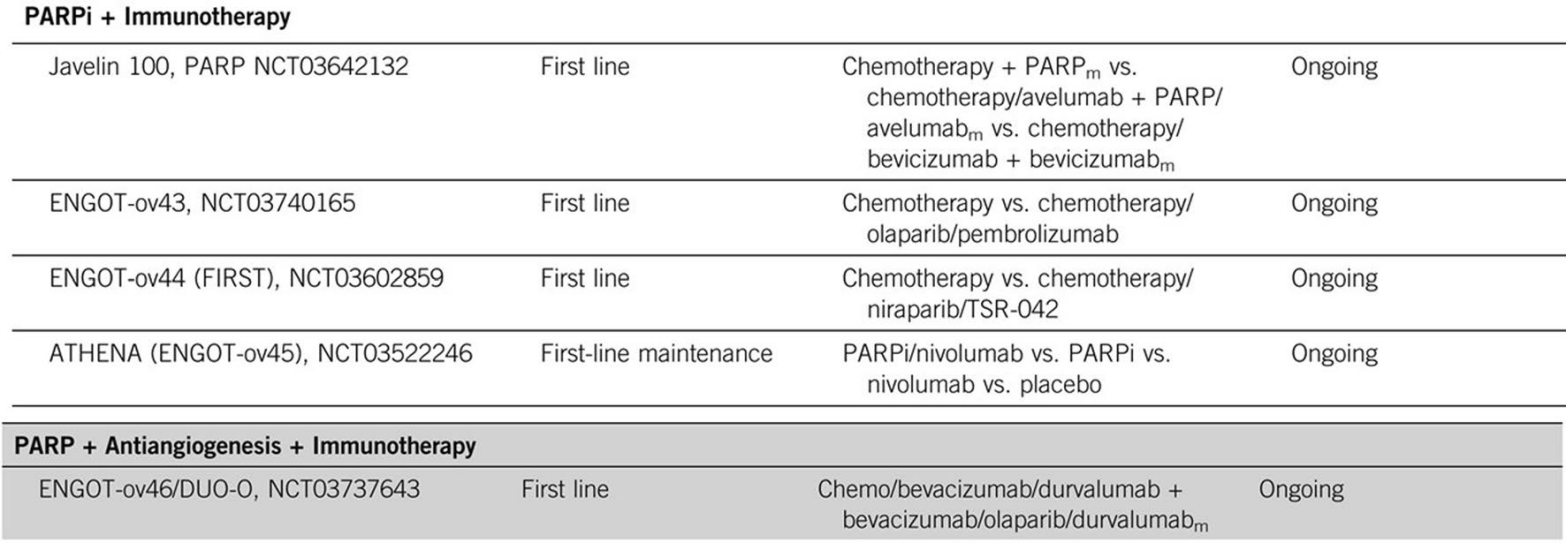
First line trials with combination strategies including PARPi + IO in ovarian cancer. Levinson et al. ASCO Ed. Book (2019)

Several trials are assessing combination strategies including immunotherapy with poly(ADP-ribose) polymerase (PARP) inhibitors, which have shown particular benefit in patients with BRCA-mutant or homologous recombination-deficient ovarian tumors (see Figs. 2, 3).

### Merkel cell carcinoma

Merkel cell carcinoma (MCC) is a rare cancer, meaning large, randomized trials are difficult to conduct. The disease is typically aggressive with poor survival and responses to chemotherapy are not durable. An early phase II trial with pembrolizumab suggested PD-1 blockade achieved clinical responses in advanced MCC in patients with and without Merkel-cell polyomavirus (MCPyV)-positive tumors [63]. A subsequent larger phase II study reported durable tumor control and favorable OS, with no significant correlation between OS or PFS and MCPyV status [64].

The first approved treatment for patients with metastatic MCC was the anti-PD-L1 antibody avelumab. In the JAVELIN Merkel 200 trial, at a median follow-up of 40.8 months, the ORR was 33% (95% CI 23.3–43.8%), including a complete response in 11.4% of patients) [65]. Median DOR was 40.5 months. Median OS was 12.6 months and the 42-month OS rate was 31%. In an updated analysis at a median follow-up of 65.1 months, median OS was 12.6 months and 5-year OS rate was 26% [66]. There was a trend for a higher response rate in patients with a high versus low tumor mutational burden (TMB), with the highest ORRs in the high TMB subgroup in patients with tumors that were also PD-L1-positive or MCPyV-negative [65]. First-line treatment with avelumab has also been associated with good responses; in 116 treatment-naïve patients treated with avelumab, the ORR was 39.7% and the durable response rate was 30.2% [67]. Avelumab was also associated with a clinically meaningful survival benefit in this cohort.

In contrast with other cancers such as melanoma, significant disease progression after cessation of PD-1/PD-L1 blockade has been observed in patients with metastatic MCC. Among 40 checkpoint inhibitor-treated patients, 14 (35%) progressed after a median follow up of 12 months from discontinuation, including those with complete responses [68]. Initial data on retreatment was promising, however, with six of eight patients having a partial or complete response.

Trials are also investigating PD-1 blockade in the neoadjuvant and adjuvant settings. In the CheckMate-358 trial, neoadjuvant nivolumab was generally tolerable and induced pathologic complete responses and radiographic tumor regressions in approximately one-half of treated patients [69]. Responses were observed regardless of tumor MCPyV, PD-L1, or TMB status. At a median follow-up of 20.3 months, median recurrence-free survival (RFS) and OS were not reached.

Treatment options are limited for patients who are not eligible for PD-1/PD-L1 inhibitors, or whose disease has progressed. Various novel strategies are being investigated for treatment of immune-checkpoint inhibitor resistant MCC, including sequential immunotherapy, or PD-1/PD-L1 blockade combined with the HDAC inhibitor domatinostat, the IL-15 superagonist N-803, and KRT‑232, a murine double minute 2 inhibitor.

## Drivers of immune responses

### Biotechnology tricks to harness CD137 (4-1BB) costimulation for cancer immunotherapy

CD137 (4-1BB) is a surface glycoprotein that belongs to the tumour necrosis factor receptor family. CD137 agonist antibodies can protect antigen-specific cytotoxic T lymphocytes from apoptosis, enhance effector function and favor persistence and memory differentiation and exert potent anticancer effects in tumor models. Anti-CD37 antibodies have also shown synergy with checkpoint blockade and ACT in preclinical models.

Two anti-CD137 antibodies have been tested in the clinical setting, urelumab and utolimumab. Initial phase I and II trials of urelumab monotherapy showed clinical activity in patients with advanced cancer but were stopped because of severe treatment-related hepatotoxicity, including two deaths [70]. Toxicity was shown to be improved via dose reduction and low doses of urelumab in combination with nivolumab have shown acceptable tolerability and promising clinical responses in patients with metastatic melanoma.

Novel strategies to revisit CD137 costimulation without severe liver inflammation include bispecific constructs designed to target CD137 costimulation specifically to the TME and direct intratumoral injection. RO7122290 is a bispecific antibody-like fusion protein based on a trimeric CD137 ligand and a targeting Fab moiety that recognizes fibroblast activation protein-α, which is expressed by cancer-associated fibroblasts in many tumors. In patients with advanced solid tumors, RO7122290 had an acceptable safety profile as monotherapy and in combination with atezolizumab [71]. Most adverse events were of grade 1/2, with no apparent dose-related pattern. Three dose-limiting toxicities were reported (grade 3 febrile neutropenia, cytokine release syndrome and pneumonitis). Preliminary antitumor activity was also shown. In blood, expression of CD137 and release of soluble CD137 indicated T-cell activation and CD137 targeting. Paired tumor biopsies revealed strong accumulation of CD8 + Ki67 + T-cells.

Another bispecific antibody, DuoBody-PD-L1 × 4-1BB (GEN1046) also demonstrated promising early activity and an acceptable safety profile in patients with advanced solid tumors in a first-in-human phase 1/2a dose escalation trial [72]. GEN1046 targets PD-L1 and CD137, simultaneously blocking the PD-L1 axis and activating T cells through conditional CD137 co-stimulation. GEN1046 demonstrated an acceptable safety profile, with the most frequent treatment-related adverse events transaminase elevations, hypothyroidism and fatigue. Transaminase elevations occurred in 26.2% of patients, with these grade 3 in 9.8% and none grade 4. Six patients experienced dose-limiting toxicities. Disease control, including stable disease at first assessment and partial responses in triple-negative breast cancer, ovarian cancer, and immune checkpoint inhibitor-pretreated non-small-cell lung cancer (NSCLC), occurred in 40 of 61 patients (65.6%). Pharmacological activity was seen across a broad range of dose levels, with increases in IFN-γ and IFN-γ–inducible protein 10 (IP-10) levels and increased frequency of proliferating total CD8 and effector memory CD8 + T cells.

**Fig. 4:**
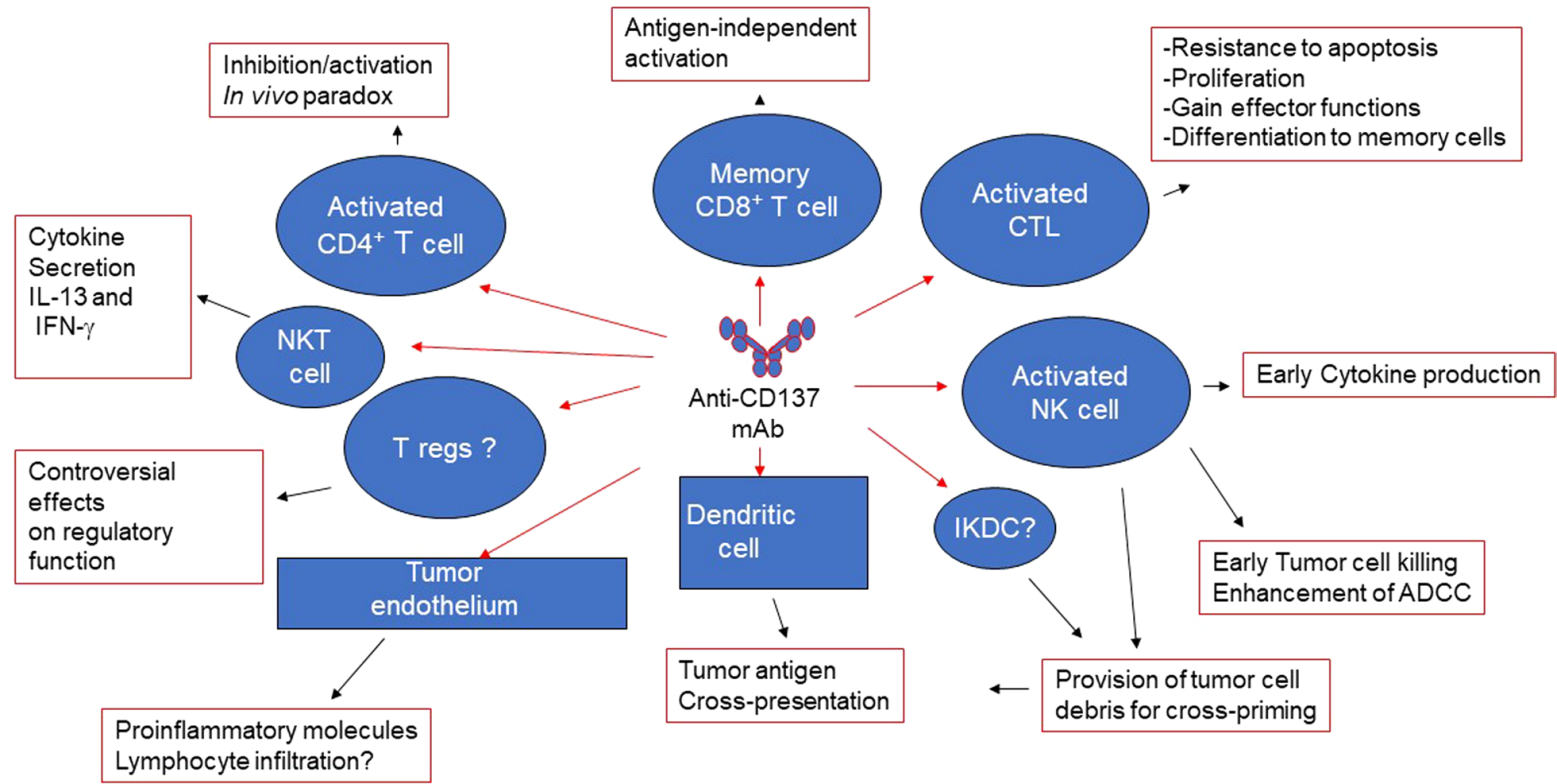
Synergy of anti-CD137 mAB

Another approach is to use a CD137 agonist antibody prodrug that is preferentially activated by tumor-associated proteases. A prodrug form of the anti-mouse CD137 agonist antibody 1D8 (1D8 Probody therapeutic, Pb-Tx) exerted antitumor effects comparable to the unmodified antibody without liver inflammation in mouse models [73]. These and other strategies to specifically target CD137 agonists to the TME are the focus of ongoing research, alongside better tolerated combination strategies, especially with checkpoint inhibitors (see Fig. 4).

### Super CD8-T cells, a new tool in immunotherapy; T cell reprogramming

Stem cell memory CD8 + T cells are highly potent and regenerative T cells. Understanding the signalling pathways that control the generation of these CD8 + T cells can provide insight into how to pharmacologically reprogram CD8 + T cells into highly effective ‘super’ T cells, thereby generating more effective immunoherapeutic approaches. Activation of CD8 T cells via the T cell receptor is usually accompanied by MEK pathway activation, which leads to persistent mitogenic stimulation, cell-cycle progression and, eventually, metabolic and T cell exhaustion. Our aim was to target this pathway to reduce T cell exhaustion, a major factor in immunotherapy failure. The addition of a MEK inhibitor to vaccination was shown to enhance antitumor response in tumor-bearing mouse models, with increased T cell infiltration, including increased antigen-specific CD8 + T cells, reduced tumor burden, and improved survival [74]. MEK inhibition prevented CD8 T cell exhaustion, with reduced markers of inhibition (CTLA-4, TIM-3, LAG-3) and increased markers of activation (CXCR3, IL-2Rβ, OX40). Metabolic fitness was enhanced by MEK inhibition, with increased mitochondrial mass in CD8 + T cells from the TME in mice. MEK inhibitor-treated ex vivo pMEL-1 cells had increased numbers of mitochondria, with higher oxygen consumption rates and lower extracellular acidification rates, indicative of enhanced mitochondrial fitness and respiratory capacity. Glucose uptake did not change with MEK inhibition; however, fatty acid uptake and fatty acid-mediated metabolism were increased. MEK inhibitor-treated T cells had increased markers of memory generation with populations of central memory-like (CD62L + CD44 +) and naive-like (CD62L + CD44-) CD8 + T cells. In the naive-like population, many cells expressed high levels of memory markers, including CD95 and CCR7, in line with recent observations of a population of minimally differentiated stem cell-like memory T cells. MEK inhibition results in CD8 + stem cell-like memory T cells with a unique metabolic profile that are distinct from naïve and central memory T cells; these cells are naive-like with lower mitochondrial potential, higher proliferative capacity, higher expression of the stem cell/T cell memory marker Sca1, higher expression of activation and memory markers, lower expression of effector and exhaustion markers, and higher expression of Klf2, which is associated with self-renewal, survival, and reduced apoptosis. MEK activation commits CD8 + T cells on a pathway to exhaustion. However, inhibiting the MEK pathway leads to CD8 + T cell reprogramming into stem cell-like memory T cells that act as a depot for effector T cells with potent therapeutic characteristics. This could be useful in enhancing CD8 + T cell products for ACT, as MEK inhibitor-treated T cells were more effective against tumors and prolonged survival in mice.

### Immunotherapy for non-immunogenic tumors

Infiltration of dendritic cells (DCs) into the TME is a key aspect of response to anti-PD-1 therapy. Overcoming resistance to immune checkpoint inhibitor therapy via DCs can involve in situ vaccination with immune modulators, mobilizing DCs to improve efficacy of immunotherapy, or the identification of immunogenic neoantigens to enhance immunity.

The TLR3 agonist poly-ICLC is an extrinsic immunomodulatory factor that has an immune stimulatory effect through activation of NK cells, macrophages and DCs, resulting in the release of cytokines and chemokines and T cell priming. In a phase I trial, therapeutic vaccination of solid cancers with intratumoral Hiltonol poly-ICLC resulted in a response rate of approximately 20%. Several trials are investigating intratumoral poly-ICLC in combination with anti-PD-1/PD-L1 antibodies.

Mobilizing and targeting DCs is another approach. Pre-treatment enhancement of responses to DC-targeted vaccination with the fms-like tyrosine kinase 3 ligand (Flt3L) was evaluated in 60 patients with high-risk melanoma, who received poly-ICLC and anti-DEC-205-NY-ESO-1 vaccine (CDX-1401), a fusion antibody targeting CD205 linked to NY-ESO-1, with or without Flt3L [75]. Antigen-specific (anti-NY-ESO-1) ex vivo T cell responses were elicited earlier, were of greater magnitude and were observed in more patients in the Flt3L arm. The majority of patients in the Flt3L arm had a positive CD8 + T cell response, while all patients with or without Flt3L had a CD4 + T cell response. Humoral immunity was of a higher magnitude and more consistently induced in Flt3L-treated patients. No evidence of epitope spreading was observed, and gene expression profiling revealed significant cell-type signatures associated with Flt3L treatment. Flt3L is now being assessed in several ongoing studies, including in combination with nivolumab in metastatic castration-resistant prostate cancer and with neoadjuvant endocrine therapy, radiation and anti-PD-1 therapy in estrogen receptor-positive breast cancer.

A third approach to overcoming resistance involves targeting immunogenic cancer neoantigens. OpenVax is a computational platform for identifying somatic variants, predicting neoantigens, and selecting the contents of personalized cancer vaccines. PGV-001 is a phase I therapeutic peptide vaccine clinical trial studying the safety and immunogenicity of a multipeptide personalized genomic vaccine for the treatment of cancers [76]. A PGV dose consists of 10 synthetic long peptides each targeting neoantigens identified from patient tumor samples together with poly-ICLC. Neoantigen selection has been feasible in all enrolled patients with adequate tumour RNA, with successful vaccine production for all these subjects. Treatment is safe and well tolerated with early validation of immunogenicity shown by CD4 + and CD8 + T cell responses. However, this type of approach involves complex manufacturing and associated costs. A shared (public) neoantigen approach may be more feasible. One example of this is mutations in the calreticulin (CALR) gene in patients with myeloproliferative neoplasms, with mutant-*CALR* eliciting antigen-specific responses from both CD4 + and CD8 + T cells, suggesting potential as a shared neoantigen [77].

### Role of dendritic cells in immune response

Cross-presentation is crucial in improving anti-tumor CD8 T cells [78]. In situ vaccination combining Flt3L, poly-ICLC, and radiotherapy induced antitumor CD8 + T cell responses in patients with advanced stage indolent NHL, some of whom had a heavy tumor burden [79]. Systemic regression of 75% was observed in some patients, with responses often durable. Systemic (abscopal) cancer remission was also observed. Non-responding patients developed a population of PD1 + CD8 + T cells, and murine tumors became newly responsive to PD-1 blockade, prompting a follow-up trial of the combined therapy [NCT03789097].

However, if in situ vaccination targets major histocompatibility complex (MHC)-I presented tumor antigens then it may be susceptible to antigen escape through relative enrichment of MHC-class I-negative tumor cells. Antigen loss is a common escape mechanism across immunotherapies. One potential approach is to target antigen-negative tumor cells via their geography and preventing antigen escape by potentiating bystander tumor killing. Using a CRISPR/Cas9 screen on a targeted library of genes highly expressed by the A20 murine lymphoma cell line identified the death receptor Fas as a critical T cell effector molecule and crucial to on-target anticancer immunity. Fas also mediates geography-dependent off-target bystander killing of antigen-negative tumor cells by antigen-specific CD8 T cells. Tumor cells can be sensitized to on-target and bystander killing by induced upregulation of Fas or inhibition of downstream regulators [80].

In murine models, CAR T induced fas-dependent on-target and bystander killing in vitro and in vivo. Fas-dependent bystander killing of antigen-negative tumors by T cells may contribute to the high response rates of antigen-directed immunotherapies despite tumoral heterogeneity. Small molecules that sensitize fas-mediated cell death independent of antigen expression, e.g., histone deacetylase (HDAC) inhibitors, may potentiate this mechanism to prevent cancer relapse.

### Pre-existing immune contexture for immunotherapy response

The Immunoscore, a routine assay quantifying CD3 + and CD8 + cytotoxic T cells within the TME, was the first immune-based classification of the host immune response [81]. It was first validated in patients with stage III colon cancer [82] and has now been incorporated into several cancer guidelines. Immunoscore might help to select patients who are most likely to benefit from longer adjuvant chemotherapy in stage III colon cancer. In patients with metastatic stage IV colon cancer, a pathological score combining clinicopathological factors for relapse with the Immunoscore was significantly associated with time to recurrence [83]. This immunopathoscore allows the stratification of stage IV patients and identification of those at higher risk of recurrence and death.

Beyond prognosis, the Immunoscore has predictive value for response to chemotherapy [84, 85]. In 763 patients with stage III colon cancer, only patients with an intermediate or high-Immunoscore responded to chemotherapy and had improved survival compared to patients not receiving chemotherapy [86]. Patients with a low Immunoscore did not significantly benefit from chemotherapy. In another study, intermediate or high Immunoscore significantly predicted benefit of six months adjuvant treatment with FOLFOX chemotherapy in clinical low-risk and high-risk stage III colon cancer [87].

In rectal cancer, a version of the Immunoscore performed on initial diagnostic biopsies (IS_B_) before neoadjuvant chemoradiotherapy can evaluate the effect of the initial immune infiltrate on both response to therapy and clinical outcome. The IS_B_ was evaluated in 249 patients with locally advanced renal cancer treated with neoadjuvant chemoradiotherapy and radical surgery. IS_B_ was positively correlated with the degree of histologic response and gene expression levels for Th1 orientation and cytotoxic immune response after neoadjuvant treatment. IS_B_ also predicted outcomes with significant differences in DFS between patients stratified by IS_B_ [88]. Importantly, IS_B_ combined with post-neoadjuvant therapy imaging was useful in identifying responders who might be candidates for an organ-preserving watch-and-wait approach. In a cohort of 73 watch-and-wait patients with complete responses after neoadjuvant chemoradiotherapy, there were no recurrence during the follow-up period in IS_B_ high patients.

### Predicting and mapping tumor regression following neoadjuvant immunotherapy

The next generation of tissue-based biomarkers will likely include the identification and quantification of multiple cell types and their spatial interaction, identified using large well-curated datasets involving multiplex and multimodal approaches. Early uses of multiplex immunofluorescence typically assessed 5–10 high power fields, but one tumor can be over 1000 fields and use over 300 GB disk space. Pathology today has parallels with astronomy 25 years ago in the need to scale and organize data, study multicolor photometry, and perform image segmentation and spatial statistics. To address these emerging pathology needs the Sloan Digital Sky Survey, a major multispectral imaging and spectroscopic astronomy tool, has been modified and adapted to host tumor-immune maps. The resultant AstroPath platform creates a framework for multiplex immunofluorescence assays and associated image analysis [89]. Use of this platform facilitated the robust assessment of the intensity of in situ PD-1 and PD-L1 expression on different cell types within the melanoma TME, with 41 combinations of expression patterns identified using six markers (PD-1, PD-L1, CD8, FoxP3, CD163, and Sox10/S100). Relatively rare cells such as CD8 + FoxP3 + cells, which represent around 2.5% of all CD8 + cells in the melanoma TME, were also mapped. These cells were highly localized to the tumor-stroma boundary and are thought to represent tumor-reactive T lymphocytes at the earliest stage after priming.

These 41 different combinations of expression patterns were next tested for an association with response and survival after anti-PD-1 therapy. In a cohort of patients with advanced, unresectable stage III/IV melanoma treated with anti-PD-1-based therapy, densities of specificCD8 + cell phenotypes, including a high density of early effector T cells characterized by CD8 + FoxP3 + PD-1^low/mid^ expression, showed strong predictive value for response, whereas a CD163 + PD-L1 − myeloid phenotype was associated with non-response. These and other key cell phenotype densities were used to develop a composite biomarker, that was highly predictive of objective response and stratified long-term patient outcomes after anti-PD-1-based therapies in both a discovery cohort and an independent validation cohort. The AstroPath platform is now being expanded to use in numerous pre-treatment and on-treatment tumor types, including in a neoadjuvant setting, and represents a unique facility for robust tissue imaging data.

### The virtual molecular tumor board as a tool for the delivery of precision oncology

Precision oncology, delivered via targeted and immune oncology therapies and guided by insights derived from tumor biomarkers, is significantly impacting patient outcomes in oncology. The clinical delivery of precision oncology is however still uneven, with evidence suggesting that currently less than half of the targeted therapy eligible patients are ever receiving a targeted therapy. Challenges are complex and importantly include a still limited use of comprehensive next generation sequencing (NGS) diagnostics, with the lack of comprehensive tumor biomarker diagnostics in the USA often associated with marked racial and socioeconomical patient disparities [90]. Clinical trial awareness and participation are also major issues.

Molecular tumor boards bring a multidisciplinary approach to cancer care, combining expertise of oncology, radiology and molecular pathology specialists in decision-making and coordination of care. Virtual molecular tumor boards take advantage of cloud-based computing, mobile device engagement, and collaborative platform software development. Software tools are now available that combine patient imaging, pathology, and automatically electronic medical record extracted clinical history information with multiomics data and treatment options, including biomarker driven clinical trials. Providence Genomics (Portland, OR, USA) aims to incorporate comprehensive genomic profiling, imaging, electronic health record content and clinical trial matching into a single data visualization and analysis software platform using an interface tailored for virtual molecular tumor boards (myPatient360). This provides a comprehensive longitudinal patient clinical timeline linked to clinical reports as well as to radiology and pathology viewers and provides a summary of biomarkers, relevant possible matched therapies and of histology stage and biomarker driven matched clinical trials. There is also the ability to search for patients with similar mutations and to compare treatments and survival curves. The multiomics database backends support genomics, epigenomics, transcriptomics, proteomics, and other data types and also provide support for summary-level features. The imaging data store combines sparse array storage (segmentations, image backgrounds) with dense storage methods and includes custom viewers for clinicians and the ability to support web-based notebook interactive computing interfaces for data analysis. This makes for a highly performant and scalable data store that is optimized for machine learning tasks. Lastly automated extraction of stage and histology via nature language processing enables biomarker-driven precise clinical trial matching and provides detailed clinical trial data extraction for review, e.g., clinical trial eligibility criteria.

We expect that the virtual molecular tumor boards based on the use of integrated data-rich patient reports with interactive will facilitate collaborative and more consistent decision-making, and also allow involvement of a wider multidisciplinary team.

### A new perspective on therapy-modulated tumor antigenicity

Although a high TMB is associated with response to immune checkpoint inhibition, of itself it is not sufficient to realize a clinical benefit. To be recognized by T cells, mutation-associated neoantigens must be synthesized at sufficient levels to be represented in MHC-I molecules expressed on the surface of tumor cells. Radiation can convert the irradiated tumor into a site for priming of tumor-specific T cells and can induce responses in tumors that are otherwise resistant to immune checkpoint blockade.

Radiation can upregulate the expression of genes that are involved in the response to DNA damage and cellular stress, thus potentially exposing immunogenic mutations to the immune system. In a poorly immunogenic mouse triple-negative breast cancer model, radiotherapy upregulated the expression of genes containing immunogenic mutations [91]. Two MHC-I and one MHC-II immunogenic neoepitopes encoded by three genes upregulated by radiotherapy were identified, DExH-box helicase 58 (Dhx58), cullin associated and neddylation dissociated 1 (Cand1), and adhesion G protein–coupled receptor F5 (Adgrf5). Vaccination with these three neoepitopes elicited polyfunctional T cell responses as well as CAND1-directed CD8 + cytotoxic T cells but did not prevent tumor growth. However, the therapeutic efficacy of radiotherapy was improved. Radiation-induced neoantigen-specific CD8 T cells preferentially killed irradiated tumor cells. Neoantigen-specific CD4 + T cells produced high levels of IFN-γ and displayed cytotoxic activity against irradiated tumor cells, which expressed low but detectable MHC-II and upregulated the cell surface death receptor Fas and DR5. These findings suggest that radiotherapy upregulates neoantigens, MHC-I and MHC-II, and death receptors, sensitizing cancer cells to killing by CD8 and CD4 T cells. Vaccination with these radiation-induced neoantigens improved control of the irradiated tumor and non-irradiated lung metastases.

Multiple types of antigens are expressed by tumors and the immunogenicity of radiation may depend on generation of signals that attract and activate antigen-presenting cells as well as diversification of the cancer neoantigen repertoire [92].

### Development of a cancer vaccine for hepatocellular carcinoma

Hepatocellular carcinoma (HCC) is a moderately mutated tumor, for which there may be immunogenic neoantigens suitable for future use in personalized therapeutic vaccination. The HepaVac Consortium (funded by EU under the FP7 program, contract nr. 602,893) has developed an “off-the-shelf” vaccine comprising multiple newly identified tumor-associated peptides naturally presented on the surface of primary HCC cells. IMA970 is a multipeptide vaccine including 12 HLA class I peptides and four HLA class II peptides combined with a novel an RNA-based immunomodulator (RNAdjuvant®). The safety, feasibility and immunogenicity of the vaccine has been tested in the multicentre HepaVac-101 phase I/II clinical trial, in which patients received a single low-dose of chemotherapy followed by nine intradermal vaccinations. The vaccine has shown a good safety profile and induction of immune responses against both class I and II peptides in this trial [93] A new HEPAVAC-201 trial protocol, including the anti-PD-L1 durvalumab, is now under evaluation.

Further identification of novel shared HCC-specific target antigens for the development of active vaccine and/or passive ACT immunotherapy strategies for HCC is ongoing. Strategies to improve antigenicity of tumor-associated antigens is also being explored, for example, through the use of heteroclitic peptides designed with specific substitutions in the residue at position 4 binding to TCRs that have higher affinity to MHC-I molecules [94, 95]

Viral infections elicit memory T-cells that may represent preventive anti-cancer vaccines. Homology between published tumor-associated antigens and non-self-viral-derived epitopes has been observed, with structural similarities between paired tumor-associated antigens and viral peptides as well as comparable patterns of contact with HLA and TCR α and β chains [96]. As such, viral antigens and tumor-associated antigens may elicit cross-reacting CD8 + T cell responses which could affect cancer progression. Previous exposure to specific viral epitopes may result in the establishment of a bispecific antiviral/anticancer T cell memory which may represent a relevant selective advantage for patients with cancer and provide a totally new set of antigens for developing a novel anticancer vaccine strategy [96].

### PDL1 and IDO targeting vaccination for treatment of metastatic cancer

Upregulation of the tryptophan-catabolizing enzyme, indoleamine 2,3-dioxygenase (IDO), suppresses T cell immunity and can be an immune escape mechanism in patients resistant to immune-checkpoint blockade. Hence, IDO represents a possible target for anticancer therapy. In a phase I trial of 15 patients with stage III-IV NSCLC treated with an IDO peptide vaccine after standard chemotherapy, median OS was 25.9 months [97]. Clinical benefit rate was 47%, with one partial response and long-lasting stable disease of ≥ 8.5 months in another six patients. After longer-term follow-up, 6-year OS rate was 20% and two of 15 patients are long-term responders with ongoing clinical responses [98]. Peripheral blood mononuclear cells from the two long-term responders showed stable CD8 + and CD4 + T-cell populations during treatment and the presence of IDO-specific T-cells.

The combination of an anti-PD-1 antibody and IDO-derived peptide vaccine could potentially increase clinical benefit, on the basis that vaccine-induced activated IDO-reactive T-cells may induce tumor PD-L1 upregulation. An ongoing randomized open-label trial is investigating the safety and efficacy of the IDO vaccine (IO102) in combination with pembrolizumab with or without chemotherapy as first-line treatment for patients with metastatic NSCLC.

In metastatic melanoma, an immune-modulatory vaccine (IO102/IO103) against IDO and PD-L1 has been evaluated in combination with nivolumab in 30 previously untreated patients in a phase I/II trial [99]. ORR was 80%, which was significantly higher than in a matched historical control group treated with anti-PD-1 monotherapy as standard of care, with 43% complete responses. At a median follow-up of 22.9 months, median PFS was 26 months and median OS was not reached. The safety profile was similar to that of nivolumab monotherapy. One patient died due to multiorgan failure related to nivolumab and two patients stopped vaccination due to injection-site granuloma or pain. There was a significant increase in vaccine-specific responses in blood detected in vitro with vaccine-reactive T cells comprising CD4 + and CD8 + T cells with activity against IDO- and PD-L1-expressing cancer and immune cells. No correlation between clinical response and vaccine induced responses were observed. IDO/PD-L1 clones increased at the tumour site in 4 of 5 patients irrespective of response. This vaccine has been granted Food and Drug Administration breakthrough therapy designation in combination with anti-PD-1 in unresectable/metastatic melanoma and a randomized trial is planned.

## Conclusions

Patients who previously had limited treatment options are now benefiting from immunotherapies that offer durable responses and improved survival. However, many patients, fail to respond to immunotherapy, especially those with less immunoresponsive cancer types, and there remains a need for new treatment strategies.

Various strategies to achieve this goal are being explored, including the development of new treatments and the combination of these and existing treatments in novel combination approaches. The efficacy of immunotherapy is largely dependent on the existence of a baseline adaptive immune response and efforts are focused on shifting the balance from an immunosuppressive TME to an immuno-activated contexture.

Immunotherapy has revolutionized the treatment of many cancers and provides a long-term survival benefit for many patients. Insights from ongoing research and further collaborative efforts, such as those summarized at this Immunotherapy Bridge, should help to continue this progress.

## Data Availability

Not applicable.

## Abbreviations

ADCC: Antibody-dependent cellular cytotoxicity
ACT: Adoptive cell transfer
ALL: Acute lymphoblastic leukemia
BLIA: Basal-like immune-activated
BLIS: Basal-like immunosuppressed
CAR: Chimeric antigen receptor
CARVac: CAR T cell Amplifying RNA Vaccine
CIS: Cytokine-inducible SH2-containing protein
CLL: Chronic lymphocytic leukemia
CPS: Combined positive score
CRS: Cytokine release syndrome
CTLA-4: Cytotoxic T-lymphocyte-associated antigen-4
DC: Dendritic cell
DCR: Disease control rate
DFS: Disease-free survival
DLBCL: Diffuse large B cell lymphoma
dMMR: Mismatch repair-deficient
DOR: Duration of response
Flt3L: Fms-like tyrosine kinase 3 ligand
HCC: Hepatocellular cancer
HDAC: Histone deacetylase
HLA: Human leukocyte antigen
HNSCC: Head and neck squamous cell carcinoma
HPV: Human papillomavirus
HR: Hazard ratio
ICANS: Immune effector cell-associated neurotoxicity syndrome
IDO: Indoleamine 2,3-dioxygenase
IFN: Interferon
IL: Interleukin
IS_B_: Immunoscore on initial diagnostic biopsy
IV: Intravenous
LAG: Lymphocyte activation gene
MCC: Merkel cell carcinoma
MCPyV: Merkel-cell polyomavirus
MHC: Major histocompatibility complex
MS/MS: Tandem mass spectrometry
MSI: Microsatellite instability
NGS: Next-generation sequencing
NHL: Non-Hodgkin lymphoma
NK: Natural killer
NSCLC: Non-small cell lung cancer
ORR: Objective response rate
OS: Overall survival
PARP: Poly(ADP-ribose) polymerase
PDAC: Pancreatic ductal adenocarcinoma
PD-1: Programmed death-1
PD-L1: Programmed death ligand-1
PFS: Progression-free survival
pIL-12-EP: Electroporated plasmid IL-12
RCC: Renal cell carcinoma
RECIST: Response Evaluation Criteria in Solid Tumors
RFS: Recurrence-free survival
TCR: T cell receptor
TGF: Transforming growth factor
Th: T helper
TIL: Tumor-infiltrating lymphocyte
TKI: Tyrosine kinase inhibitor
TLR: Toll-like receptor
TMB: Tumor mutational burden
TME: Tumor microenvironment
TNBC: Triple-negative breast cancer
TNF: Tumor necrosis factor
Tregs: Regulatory T cells
VEGF: Vascular endothelial growth factor

## References

[1] Nelson MH, Knochelmann HM, Bailey SR, Huff LW, Bowers JS, Majchrzak-Kuligowska K (2020). Identification of human CD4+ T cell populations with distinct antitumor activity. Sci Adv.

[2] Knochelmann HM, Horton JD, Liu S, Armeson K, Kaczmar JM, Wyatt MM (2021). Neoadjuvant presurgical PD-1 inhibition in oral cavity squamous cell carcinoma. Cell Rep Med.

[3] Liu S, Knochelmann HM, Lomeli SH, Hong A, Richardson M, Yang Z (2021). Response and recurrence correlates in individuals treated with neoadjuvant anti-PD-1 therapy for resectable oral cavity squamous cell carcinoma. Cell Rep Med.

[4] Klebanoff CA, Wolchok JD (2018). Shared cancer neoantigens: making private matters public. J Exp Med.

[5] Priestley P, Baber J, Lolkema MP, Steeghs N, de Bruijn E, Shale C (2019). Pan-cancer whole-genome analyses of metastatic solid tumours. Nature.

[6] Chandran SS, Ma J, Klatt MG, Dündar F, Bandlamudi C, Razavi P, Wen HY (2022). Immunogenicity and therapeutic targeting of a public neoantigen derived from mutated PIK3CA. Nat Med.

[7] Chandran SS, Klebanoff CA (2019). T cell receptor-based cancer immunotherapy: emerging efficacy and pathways of resistance. Immunol Rev.

[8] Liu E, Marin D, Banerjee P, Macapinlac HA, Thompson P, Basar R (2020). Use of CAR-transduced natural killer cells in CD19-positive lymphoid tumors. N Engl J Med.

[9] Delconte RB, Kolesnik TB, Dagley LF, Rautela J, Shi W, Putz EM (2016). CIS is a potent checkpoint in NK cell-mediated tumor immunity. Nat Immunol.

[10] Zhu H, Blum RH, Bernareggi D, Ask EH, Wu Z (2020). Metabolic reprograming via deletion of CISH in human iPSC-derived NK cells promotes in vivo persistence and enhances anti-tumor activity. Cell Stem Cell.

[11] Daher M, Rezvani K (2021). Outlook for new CAR-based therapies with a focus on CAR NK cells: what lies beyond CAR-engineered T cells in the race against cancer. Cancer Discov.

[12] Cerrano M, Ruella M, Perales MA, Vitale C, Faraci DG, Giaccone L (2020). The advent of CAR T-cell therapy for lymphoproliferative neoplasms: integrating research into clinical practice. Front Immunol.

[13] Ghilardi G, Braendstrup P, Chong EA, Schuster SJ, Svoboda J, Ruella M (2021). CAR-T TREK through the lymphoma universe, to boldly go where no other therapy has gone before. Br J Haematol.

[14] Ruella M, Maus MV (2016). Catch me if you can: leukemia escape after CD19-directed T cell immunotherapies. Comput Struct Biotechnol J.

[15] Maude SL, Laetsch TW, Buechner J, Rives S, Boyer M, Bittencourt H (2018). Tisagenlecleucel in children and young adults with B-cell lymphoblastic leukemia. N Engl J Med.

[16] Spiegel JY, Patel S, Muffly L, Hossain NM, Oak J, Baird JH (2021). CAR T cells with dual targeting of CD19 and CD22 in adult patients with recurrent or refractory B cell malignancies: a phase 1 trial. Nat Med.

[17] Frey NV, Gill S, Hwang W-T, Luger SM, Martin ME, McCurdy SR (2021). CART22-65s co-administered with huCART19 in adult patients with relapsed or refractory all. Blood.

[18] Schuster SJ, Bartlett NL, Assouline S, Yoon S-S, Bosch F, Sehn LH (2019). Mosunetuzumab induces complete remissions in poor prognosis non-hodgkin lymphoma patients, including those who are resistant to or relapsing after chimeric antigen receptor T-cell (CAR-T) therapies, and is active in treatment through multiple lines. Blood.

[19] Smith M, Dai A, Ghilardi G, Amelsberg KV, Devlin SM, Pajarillo R (2022). Gut microbiome correlates of response and toxicity following anti-CD19 CAR T cell therapy. Nat Med.

[20] Gill SI, Frey NV, Hexner E, Metzger S, O'Brien M, Hwang WT (2022). Anti-CD19 CAR T cells in combination with ibrutinib for the treatment of chronic lymphocytic leukemia. Blood Adv.

[21] Rodas IM, Saiag P, Merino LDLC, Dutriaux C, Rodríguez-Moreno J, Robert C (2021). Preliminary results of a phase 2 study of intratumoral administration of BO-112 with pembrolizumab in patients with advanced melanoma that have progressive disease on anti-PD-1-based therapy. J Immunother Cancer.

[22] Tselikas L, Robert C, Dalle S, Meyer N, Lebbe C, Ammari S (2019). Safety and efficacy of intratumoral ipilimumab with IV nivolumab in metastatic melanoma. The NIVIPIT trial. J Immunother Cancer.

[23] Kirkwood J, Zakharia Y, Davar D, Buchbinder E, Medina T, Daud A (2021). Final analysis: phase 1b study investigating intratumoral injection of toll-like receptor 9 agonist vidutolimod ± pembrolizumab in patients with PD-1 blockade–refractory melanoma. J Immunother Cancer.

[24] Fernandez-Penas P, Carlino M, Tsai K, Atkinson V, Shaheen M, Thomas S (2021). Durable responses with intratumoral electroporation of plasmid interleukin 12 plus pembrolizumab in patients with advanced melanoma progressing on an anti-PD-1 antibody: updated data from KEYNOTE 695. J Immunother Cancer.

[25] Weiss S, Sznol M, Shaheen M, Berciano-Guerrero M-A, Felip E, Rodríguez-Abreu D (2021). Phase II of CD40 agonistic antibody sotigalimab (APX005M) in combination with nivolumab in subjects with metastatic melanoma with confirmed disease progression on anti-PD-1 therapy. J Immunother Cancer.

[26] Loquai C, Hassel J, Brück P, Derhovanessian E, Cuk K, Lörks V (2021). An RNA-lipoplex (RNA-LPX) vaccine demonstrates strong immunogenicity and promising clinical activity in a phase I trial in cutaneous melanoma patients with no evidence of disease at trial inclusion. J Immunother Cancer.

[27] Mackensen A, Koenecke C, Haanen J, Alsdorf W, Desuki A, Wagner-Drouet E (2021). BNT211: a phase I/II trial to evaluate safety and efficacy of CLDN6 CAR-T cells and vaccine-mediated in vivo expansion in patients with CLDN6-positive advanced solid tumors. J Immunother Cancer.

[28] Ferris RL, Blumenschein G, Fayette J, Guigay J, Colevas AD, Licitra L (2016). Nivolumab for recurrent squamous-cell carcinoma of the head and neck. N Engl J Med.

[29] Ferris RL, Blumenschein G, Fayette J, Guigay J, Colevas AD, Licitra L (2018). Nivolumab vs investigator's choice in recurrent or metastatic squamous cell carcinoma of the head and neck: 2-year long-term survival update of CheckMate 141 with analyses by tumor PD-L1 expression. Oral Oncol.

[30] Burtness B, Harrington KJ, Greil R, Soulières D, Tahara M, de Castro Jr G (2019). Pembrolizumab alone or with chemotherapy versus cetuximab with chemotherapy for recurrent or metastatic squamous cell carcinoma of the head and neck (KEYNOTE-048): a randomised, open-label, phase 3 study. Lancet.

[31] Lee NY, Ferris RL, Psyrri A, Haddad RI, Tahara M, Bourhis J (2021). Avelumab plus standard-of-care chemoradiotherapy versus chemoradiotherapy alone in patients with locally advanced squamous cell carcinoma of the head and neck: a randomised, double-blind, placebo-controlled, multicentre, phase 3 trial. Lancet Oncol.

[32] Ferris RL, Spanos WC, Leidner R, Gonçalves A, Martens UM, Kyi C (2021). Neoadjuvant nivolumab for patients with resectable HPV-positive and HPV-negative squamous cell carcinomas of the head and neck in the CheckMate 358 trial. J Immunother Cancer.

[33] Mascarella MA, Vendra V, Kubik M, Sridharan S, Kim S, Ferris RL (2021). Effect of neoadjuvant systemic therapy given during window trials on quality metrics in resectable head and neck squamous cell carcinoma. J Clin Oncol.

[34] Schoenfeld JD, Hanna GJ, Jo VY, Rawal B, Chen YH, Catalano PS (2020). Neoadjuvant nivolumab or nivolumab plus ipilimumab in untreated oral cavity squamous cell carcinoma: a phase 2 open-label randomized clinical trial. JAMA Oncol.

[35] Albiges L, Tannir NM, Burotto M, McDermott D, Plimack ER, Barthélémy P (2020). Nivolumab plus ipilimumab versus sunitinib for first-line treatment of advanced renal cell carcinoma: extended 4-year follow-up of the phase III CheckMate 214 trial. ESMO Open.

[36] Motzer RJ, Tannir NM, McDermott DF, Burotto M, Choueiri TK, Hammers HJ (2021). Conditional survival and 5-year follow-up in CheckMate 214: First-line nivolumab + ipilimumab (N+I) versus sunitinib (S) in advanced renal cell carcinoma (aRCC). Ann Oncol.

[37] Choueiri TK, Powles T, Burotto M, Escudier B, Bourlon MT, Zurawski B (2021). Nivolumab plus cabozantinib versus sunitinib for advanced renal-cell carcinoma. N Engl J Med.

[38] Rini BI, Plimack ER, Stus V, Gafanov R, Hawkins R, Nosov D (2019). Pembrolizumab plus axitinib versus sunitinib for advanced renal-cell carcinoma. N Engl J Med.

[39] Rini BI, Plimack ER, Stus V, Waddell T, Gafanov R, Pouliot F (2021). Pembrolizumab (pembro) plus axitinib (axi) versus sunitinib as first-line therapy for advanced clear cell renal cell carcinoma (ccRCC): Results from 42-month follow-up of KEYNOTE-426. J Clin Oncol.

[40] Motzer R, Alekseev B, Rha SY, Porta C, Eto M, Powles T (2021). Lenvatinib plus pembrolizumab or everolimus for advanced renal cell carcinoma. N Engl J Med.

[41] Choueiri TK, Tomczak P, Park SH, Venugopal B, Ferguson T, Chang YH (2021). Adjuvant pembrolizumab after nephrectomy in renal-cell carcinoma. N Engl J Med.

[42] Kato K, Cho BC, Takahashi M, Okada M, Lin CY, Chin K (2019). Nivolumab versus chemotherapy in patients with advanced oesophageal squamous cell carcinoma refractory or intolerant to previous chemotherapy (ATTRACTION-3): a multicentre, randomised, open-label, phase 3 trial. Lancet Oncol.

[43] Kojima T, Shah MA, Muro K, Francois E, Adenis A, Hsu CH (2020). Randomized phase III KEYNOTE-181 study of pembrolizumab versus chemotherapy in advanced esophageal cancer. J Clin Oncol.

[44] Sun JM, Shen L, Shah MA, Enzinger P, Adenis A, Doi T (2021). Pembrolizumab plus chemotherapy versus chemotherapy alone for first-line treatment of advanced oesophageal cancer (KEYNOTE-590): a randomised, placebo-controlled, phase 3 study. Lancet.

[45] Doki Y, Ajani JA, Kato K, Xu J, Wyrwicz L, Motoyama S (2022). Nivolumab combination therapy in advanced esophageal squamous-cell carcinoma. N Engl J Med.

[46] Luo H, Lu J, Bai Y, Mao T, Wang J, Fan Q (2021). Effect of camrelizumab vs placebo added to chemotherapy on survival and progression-free survival in patients with advanced or metastatic esophageal squamous cell carcinoma: the ESCORT-1st randomized clinical trial. JAMA.

[47] Janjigian YY, Shitara K, Moehler M, Garrido M, Salman P, Shen L (2021). First-line nivolumab plus chemotherapy versus chemotherapy alone for advanced gastric, gastro-oesophageal junction, and oesophageal adenocarcinoma (CheckMate 649): a randomised, open-label, phase 3 trial. Lancet.

[48] Janjigian YY, Ajani JA, Moehler M (2021). Nivolumab plus chemotherapy or ipilimumab vs chemotherapy as first-line treatment for advanced gastric cancer/gastroesophageal junction cancer/esophageal adenocarcinoma: CheckMate 649 study. Ann Oncol.

[49] Kelly RJ, Ajani JA, Kuzdzal J, Zander T, Van Cutsem E, Piessen G (2021). Adjuvant nivolumab in resected esophageal or gastroesophageal junction cancer. N Engl J Med.

[50] André T, Shiu KK, Kim TW, Jensen BV, Jensen LH, Punt C (2020). Pembrolizumab in microsatellite-instability-high advanced colorectal cancer. N Engl J Med.

[51] Avallone A, De Stefano A, Pace U, Catteau A, Di Gennaro E, Tatangelo F (2020). Neoadjuvant nivolumab in early stage colorectal cancer. Ann Oncol.

[52] Chalabi M, Fanchi LF, Dijkstra KK, Van den Berg JG, Aalbers AG, Sikorska K (2020). Neoadjuvant immunotherapy leads to pathological responses in MMR-proficient and MMR-deficient early-stage colon cancers. Nat Med.

[53] Emens LA, Adams S, Barrios CH, Diéras V, Iwata H, Loi S (2021). First-line atezolizumab plus nab-paclitaxel for unresectable, locally advanced, or metastatic triple-negative breast cancer: IMpassion130 final overall survival analysis. Ann Oncol.

[54] Emens LA, Cruz C, Eder JP, Braiteh F, Chung C, Tolaney SM (2019). Long-term clinical outcomes and biomarker analyses of atezolizumab therapy for patients with metastatic triple-negative breast cancer: a phase 1 study. JAMA Oncol.

[55] Emens LA, Molinero L, Loi S, Rugo HS, Schneeweiss A, Diéras V (2021). Atezolizumab and nab-paclitaxel in advanced triple-negative breast cancer: biomarker evaluation of the IMpassion130 study. J Natl Cancer Inst.

[56] Emens LA, Goldstein LD, Schmid P, Rugo HS, Adams S, Barrios CH (2021). The tumor microenvironment (TME) and atezolizumab + nab-paclitaxel (A+nP) activity in metastatic triple-negative breast cancer (mTNBC): IMpassion130. J Clin Oncol.

[57] Hwang WT, Adams SF, Tahirovic E, Hagemann IS, Coukos G (2012). Prognostic significance of tumor-infiltrating T cells in ovarian cancer: a meta-analysis. Gynecol Oncol.

[58] Webb JR, Milne K, Kroeger DR, Nelson BH (2016). PD-L1 expression is associated with tumor-infiltrating T cells and favorable prognosis in high-grade serous ovarian cancer. Gynecol Oncol.

[59] Omatsu K, Hamanishi J, Katsumata N, Nishio S, Sawada K, Takeuchi S (2020). 807O - Nivolumab versus gemcitabine or pegylated liposomal doxorubicin for patients with platinum-resistant (advanced or recurrent) ovarian cancer: open-label, randomized trial in Japan (NINJA trial). Ann Oncol.

[60] Pujade-Lauraine E, Fujiwara K, Ledermann JA, Oza AM, Kristeleit R, Ray-Coquard IL (2021). Avelumab alone or in combination with chemotherapy versus chemotherapy alone in platinum-resistant or platinum-refractory ovarian cancer (JAVELIN Ovarian 200): an open-label, three-arm, randomised, phase 3 study. Lancet Oncol.

[61] Monk BJ, Colombo N, Oza AM, Fujiwara K, Birrer MJ, Randall L (2021). Chemotherapy with or without avelumab followed by avelumab maintenance versus chemotherapy alone in patients with previously untreated epithelial ovarian cancer (JAVELIN Ovarian 100): an open-label, randomised, phase 3 trial. Lancet Oncol.

[62] Moore KN, Bookman M, Sehouli J, Miller A, Anderson C, Scambia G (2021). Atezolizumab, bevacizumab, and chemotherapy for newly diagnosed stage III or IV ovarian cancer: placebo-controlled randomized phase III trial (IMagyn050/GOG 3015/ENGOT-OV39). J Clin Oncol.

[63] Nghiem PT, Bhatia S, Lipson EJ, Kudchadkar RR, Miller NJ, Annamalai L (2016). PD-1 blockade with pembrolizumab in advanced merkel-cell carcinoma. N Engl J Med.

[64] Nghiem P, Bhatia S, Lipson EJ, Sharfman WH, Kudchadkar RR, Brohl AS (2019). Durable tumor regression and overall survival in patients with advanced merkel cell carcinoma receiving pembrolizumab as first-line therapy. J Clin Oncol.

[65] D'Angelo SP, Bhatia S, Brohl AS, Hamid O, Mehnert JM, Terheyden P (2020). Avelumab in patients with previously treated metastatic Merkel cell carcinoma: long-term data and biomarker analyses from the single-arm phase 2 JAVELIN Merkel 200 trial. J Immunother Cancer.

[66] Nghiem P, Bhatia S, Brohl AS, Hamid O, Mehnert JM, Terheyden P (2021). Avelumab in patients with previously treated Merkel cell carcinoma (JAVELIN Merkel 200): Updated overall survival data after more than five years of follow up. J Clin Oncol.

[67] D'Angelo SP, Lebbé C, Mortier L, Brohl AS, Fazio N, Grob JJ (2021). First-line avelumab in a cohort of 116 patients with metastatic Merkel cell carcinoma (JAVELIN Merkel 200): primary and biomarker analyses of a phase II study. J Immunother Cancer.

[68] Weppler AM, Da Meda L, Silva I, Xu W, Grignani G, Menzies AM (2021). Durability of response to immune checkpoint inhibitors (ICI) in metastatic Merkel cell carcinoma (mMCC) after treatment cessation. J Clin Oncol.

[69] Topalian SL, Bhatia S, Amin A, Kudchadkar RR, Sharfman WH, Lebbé C (2020). Neoadjuvant nivolumab for patients With resectable merkel cell carcinoma in the checkmate 358 trial. J Clin Oncol.

[70] Segal NH, Logan TF, Hodi FS, McDermott D, Melero I, Hamid O (2017). Results from an integrated safety analysis of urelumab, an agonist anti-CD137 monoclonal antibody. Clin Cancer Res.

[71] Melero I, Sanmamed MF, Calvo E, Moreno I, Moreno V, Hernandez Guerrero TC (2020). 1025MO first-in-human (FIH) phase I study of RO7122290 (RO), a novel FAP-targeted 4–1BB agonist, administered as single agent and in combination with atezolizumab (ATZ) to patients with advanced solid tumours. Ann Oncol.

[72] Garralda E, Geva R, Ben-Ami E, Maurice-Dror C, Calvo E, LoRusso P (2020). First-in-human phase I/IIa trial to evaluate the safety and initial clinical activity of DuoBody®-PD-L1×4–1BB (GEN1046) in patients with advanced solid tumors. J Immunother Cancer.

[73] Etxeberria I, Bolaños E, Teijeira A, Garasa S, Yanguas A, Azpilikueta A (2021). Antitumor efficacy and reduced toxicity using an anti-CD137 Probody therapeutic. Proc Natl Acad Sci USA.

[74] Verma V, Jafarzadeh N, Boi S, Kundu S, Jiang Z, Fan Y (2021). MEK inhibition reprograms CD8+ T lymphocytes into memory stem cells with potent antitumor effects. Nat Immunol.

[75] Bhardwaj N, Friedlander PA, Pavlick AC, Ernstoff MS, Gastman BR, Hanks BA (2020). Flt3 ligand augments immune responses to anti-DEC-205-NY-ESO-1 vaccine through expansion of dendritic cell subsets. Nat Cancer.

[76] Rubinsteyn A, Kodysh J, Hodes I, Mondet S, Aksoy BA, Finnigan JP (2018). Computational pipeline for the PGV-001 neoantigen vaccine trial. Front Immunol.

[77] Cimen Bozkus C, Roudko V, Finnigan JP, Mascarenhas J, Hoffman R, Iancu-Rubin C, Bhardwaj N (2019). Immune checkpoint blockade enhances shared neoantigen-induced T-cell immunity directed against mutated calreticulin in myeloproliferative neoplasms. Cancer Discov.

[78] Lubitz GS, Brody JD (2022). Not just neighbours: positive feedback between tumour-associated macrophages and exhausted T cells. Nat Rev Immunol.

[79] Hammerich L, Marron TU, Upadhyay R, Svensson-Arvelund J, Dhainaut M, Hussein S (2019). Systemic clinical tumor regressions and potentiation of PD1 blockade with in situ vaccination. Nat Med.

[80] Upadhyay R, Boiarsky JA, Pantsulaia G, Svensson-Arvelund J, Lin MJ, Wroblewska A (2021). A critical role for fas-mediated off-target tumor killing in T-cell immunotherapy. Cancer Discov.

[81] Bindea G, Mlecnik B, Fridman WH, Galon J (2011). The prognostic impact of anti-cancer immune response: a novel classification of cancer patients. Semin Immunopathol.

[82] Pagès F, Mlecnik B, Marliot F, Bindea G, Ou FS, Bifulco C (2018). International validation of the consensus immunoscore for the classification of colon cancer: a prognostic and accuracy study. Lancet.

[83] Baldin P, Van den Eynde M, Mlecnik B, Galon J (2020). Immunity to live: an immunopathoscore using the consensus immunoscore to best define the risk of recurrence and death in stage IV metastatic patients. Oncoimmunology.

[84] Bindea G, Mlecnik B, Angell HK, Galon J (2014). The immune landscape of human tumors: implications for cancer immunotherapy. Oncoimmunology.

[85] Ascierto PA, Marincola FM, Fox BA, Galon J (2020). No time to die: the consensus immunoscore for predicting survival and response to chemotherapy of locally advanced colon cancer patients in a multicenter international study. Oncoimmunology.

[86] Mlecnik B, Bifulco C, Bindea G, Marliot F, Lugli A, Lee JJ (2020). Multicenter international society for immunotherapy of cancer study of the consensus immunoscore for the prediction of survival and response to chemotherapy in stage III colon cancer. J Clin Oncol.

[87] Pagès F, André T, Taieb J, Vernerey D, Henriques J, Borg C (2020). Prognostic and predictive value of the immunoscore in stage III colon cancer patients treated with oxaliplatin in the prospective IDEA France PRODIGE-GERCOR cohort study. Ann Oncol.

[88] El Sissy C, Kirilovsky A, Van den Eynde M, Muşină AM, Anitei MG, Romero A (2020). A diagnostic biopsy-adapted immunoscore predicts response to neoadjuvant treatment and selects patients with rectal cancer eligible for a watch-and-wait strategy. Clin Cancer Res.

[89] Berry S, Giraldo NA, Green BF, Cottrell TR, Stein JE, Engle EL (2021). Analysis of multispectral imaging with the AstroPath platform informs efficacy of PD-1 blockade. Science.

[90] Bruno DS, Hess LM, Li X, Su EW, Zhu YE, Patel M (2021). Racial disparities in biomarker testing and clinical trial enrollment in non-small cell lung cancer (NSCLC). J Clin Oncol.

[91] Lhuillier C, Rudqvist NP, Yamazaki T, Zhang T, Charpentier M, Galluzzi L (2021). Radiotherapy-exposed CD8+ and CD4+ neoantigens enhance tumor control. J Clin Invest.

[92] Lhuillier C, Rudqvist NP, Elemento O, Formenti SC, Demaria S (2019). Radiation therapy and anti-tumor immunity: exposing immunogenic mutations to the immune system. Genome Med.

[93] Löffler (2022). Phase I/II multicenter trial of a novel therapeutic cancer vaccine, HepaVac-101, for hepatocellular carcinoma. Clin Cancer Res.

[94] Cavalluzzo B, Ragone C, Mauriello A, Petrizzo A, Manolio C, Caporale A (2021). Identification and characterization of heteroclitic peptides in TCR-binding positions with improved HLA-binding efficacy. J Transl Med.

[95] Tagliamonte M (2021). MHC-optimized peptide scaffold for improved antigen presentation and anti-tumor response. Front Immunol.

[96] Ragone C, Manolio C, Cavalluzzo B, Mauriello A, Tornesello ML, Buonaguro FM (2021). Identification and validation of viral antigens sharing sequence and structural homology with tumor-associated antigens (TAAs). J Immunother Cancer.

[97] Iversen TZ, Engell-Noerregaard L, Ellebaek E, Andersen R, Larsen SK, Bjoern J (2014). Long-lasting disease stabilization in the absence of toxicity in metastatic lung cancer patients vaccinated with an epitope derived from indoleamine 2,3 dioxygenase. Clin Cancer Res.

[98] Kjeldsen JW, Iversen TZ, Engell-Noerregaard L, Mellemgaard A, Andersen MH, Svane IM (2018). Durable clinical responses and long-term follow-up of stage III-IV non-small-cell lung cancer (NSCLC) patients treated with IDO peptide vaccine in a phase I study-a brief research report. Front Immunol.

[99] Kjeldsen JW, Lorentzen CL, Martinenaite E, Ellebaek E, Donia M, Holmstroem RB (2021). A phase 1/2 trial of an immune-modulatory vaccine against IDO/PD-L1 in combination with nivolumab in metastatic melanoma. Nat Med.

